# Prospects for targeting ACKR1 in cancer and other diseases

**DOI:** 10.3389/fimmu.2023.1111960

**Published:** 2023-03-15

**Authors:** Kyler S. Crawford, Brian F. Volkman

**Affiliations:** Department of Biochemistry, Medical College of Wisconsin, Milwaukee, WI, United States

**Keywords:** ACKR1, DARC, chemokine, cancer, inflammation

## Abstract

The chemokine network is comprised of a family of signal proteins that encode messages for cells displaying chemokine G-protein coupled receptors (GPCRs). The diversity of effects on cellular functions, particularly directed migration of different cell types to sites of inflammation, is enabled by different combinations of chemokines activating signal transduction cascades on cells displaying a combination of receptors. These signals can contribute to autoimmune disease or be hijacked in cancer to stimulate cancer progression and metastatic migration. Thus far, three chemokine receptor-targeting drugs have been approved for clinical use: Maraviroc for HIV, Plerixafor for hematopoietic stem cell mobilization, and Mogalizumab for cutaneous T-cell lymphoma. Numerous compounds have been developed to inhibit specific chemokine GPCRs, but the complexity of the chemokine network has precluded more widespread clinical implementation, particularly as anti-neoplastic and anti-metastatic agents. Drugs that block a single signaling axis may be rendered ineffective or cause adverse reactions because each chemokine and receptor often have multiple context-specific functions. The chemokine network is tightly regulated at multiple levels, including by atypical chemokine receptors (ACKRs) that control chemokine gradients independently of G-proteins. ACKRs have numerous functions linked to chemokine immobilization, movement through and within cells, and recruitment of alternate effectors like β-arrestins. Atypical chemokine receptor 1 (ACKR1), previously known as the Duffy antigen receptor for chemokines (DARC), is a key regulator that binds chemokines involved in inflammatory responses and cancer proliferation, angiogenesis, and metastasis. Understanding more about ACKR1 in different diseases and populations may contribute to the development of therapeutic strategies targeting the chemokine network.

## Introduction

Chemokine receptors (CKRs) are specialized seven-transmembrane domain surface receptors in the class A subfamily of the G-protein coupled receptor (GPCR) superfamily. Chemokine ligands are small, structurally-conserved proteins categorized by the configuration of a cysteine motif (CXC, CC, CX3C, C) in the N-terminus (1). The classical function of chemokine GPCRs is to activate leukocyte migration along increasing chemokine concentration gradients towards their source, with different tissues producing distinct combinations of chemokines to attract specific cell types. Chemokine messages elicit complex, multicellular responses encoded in the combinatorial diversity of overlapping ligand-receptor specificities and dynamic membrane interactions. Receptor stimulation recruits β-arrestins, an intracellular effector that decreases activation of heterotrimeric G-proteins, scaffolds cytoskeletal adaptors that internalize surface receptors, and signals through distinct pathways (2). The chemokine network is tightly regulated with overlapping mechanisms to amplify, diversify, and resolve cellular signals (3). One arm of chemokine control is exerted through expression of atypical chemokine receptors (ACKRs), dedicated chemokine receptors uncoupled from G-protein cascades that regulate chemokine patterning and GPCR sensitivity (4). CKRs and ACKRs have complementary roles in exerting and modulating chemokine function. ACKRs have an independent role to bind, scavenge, and traffic chemokine ligands and maintain gradients so that cells are directed to their functional compartments (5). ACKRs can also directly regulate GPCR signaling through ligand depletion or resolution of activated intracellular cascades.

Chemokine signals are crucial for immune cell recruitment, embryonic development, and retention of discrete cellular niches. Consequently, dysregulation of the chemokine network can contribute to a multitude of disease and CKRs are appealing therapeutic drug targets. GPCRs are the target of a third or more of all drugs, but chemokine GPCRs present unique challenges to drug design that prevent compounds from progressing to approved therapeutics (6, 7). Inhibitors of individual GPCRs can have deleterious side effects by perturbing the balance of these signal pathways and interfering in unrelated physiological functions that involve the target GPCR. A druggable chemokine network becomes more achievable when the interplay of signaling and regulatory components in the system is well-understood. Here we discuss the role of ACKR1/DARC in disease and potential therapeutic strategies.

## The atypical chemokine receptor family

**Figure 1:**
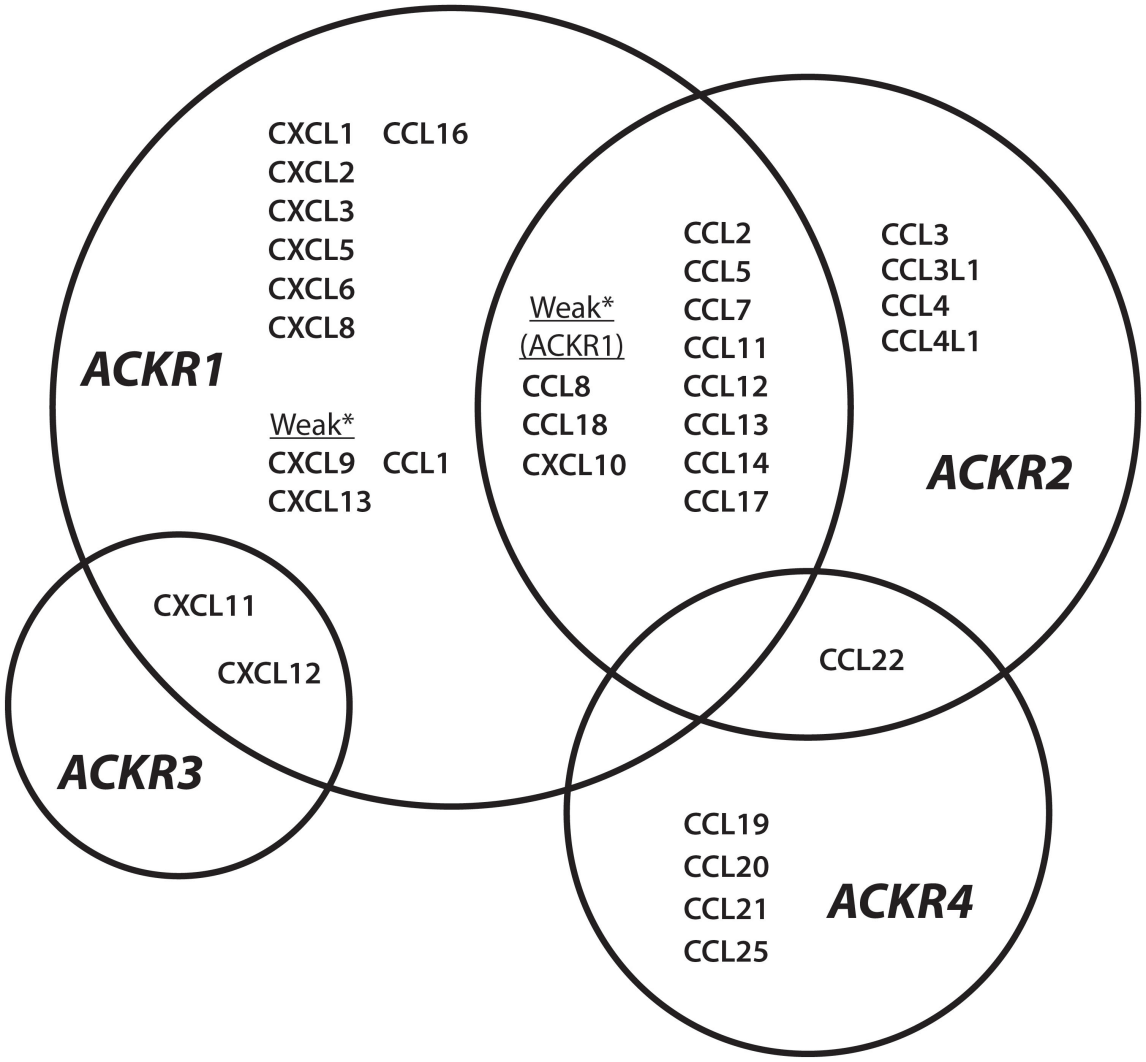
ACKR1-4 chemokine interaction network Chemokine ligands described for atypical chemokine receptors ACKR1, ACKR2, ACKR3, and ACKR4. *Chemokines are described as weak binders to ACKR1.

The four known atypical chemokine receptors, ACKR1-4, exhibit distinct expression patterns, chemokine-binding profiles, and cellular effects. The chemokine ligands of the atypical receptors are shown in **Figure 1**. ACKR1 is a promiscuous receptor for chemokines involved in diverse functions including angiogenesis, chemotaxis, and cellular retention signals. Expression is restricted to erythroid cells, cerebellar Purkinje neurons and the endothelial cell lining of capillary-draining venules, where ACKR1 binds and transports chemokines. ACKR2 binds the second-most chemokines and was thought to be restricted to binding CC-class chemokines until recent reports have described interactions with CXCL10 and CXCL14 (8–10). ACKR2 is primarily found in the lymphatic, not vascular, endothelium but it is also expressed in certain B-lymphocytes, myeloid immune cells, and developing trophoblasts (11–13). ACKR2 serves as a chemokine scavenger that constitutively recycles from membrane to endosome through a pathway involving β-arrestin (14). ACKR3 is a high affinity receptor for several proteins including endogenous opioid peptides and viral chemokine vCCL2/vMIP-II, but only binds two human chemokines, CXCL11 and CXCL12 (15, 16). ACKR3 expression has been described in a diversity of cell types with increasing evidence of ligand-specific, β-arrestin-mediated signaling pathways and multiple internalization mechanisms (17–19). ACKR4 binds CCL19, CCL20, CCL21, CCL22 and CCL25, a subset of chemokines associated with spatial organization of T-cells and dendritic cells (20). Knowledge of ACKR4 expression is incomplete, but it has been characterized as a component of endothelial barriers in tissues including the skin, spleen, and lymphatic vasculature and as a scavenger on fibroblasts in the dermis and intestinal submucosa (21–23). ACKR4 scavenging uses a similar internalization mechanism to ACKR2 involving β-arrestin recruitment, but without the downstream ERK1/2, Akt, or Src kinase activation attributed to ACKR3 (24). Candidate members of the ACKR family include CC chemokine receptor-like 2 (CCRL2/ACKR5) as a receptor for the chemotactic protein chemerin, and membrane-associated phosphatidylinositol transfer protein 3 (PITPNM3/ACKR6) as a receptor for CCL18 (25, 26). Overall, ACKRs bind the majority of CC and CXC chemokines and expression is spatially organized in tissues to maintain functional chemokine gradients and regulate GPCR signaling. ACKR1 has several advantages as a potential drug target because it is promiscuous and encompasses multiple important chemokine-induced pathways, while being uncoupled from direct signal transduction and exhibiting restricted tissue expression. 

### ACKR1 genetics

ACKR1 expression in humans was initially described as the “Duffy” or “Fy” blood group after a hemophiliac patient who developed hemolytic reactions from mismatched blood (27). The recognition sites of the “Fy-reactive” antibodies were mapped to distinct erythrocyte surface antigens, later revealed to correspond to regions of ACKR1. These include a conformational epitope (Fy3) capturing the extracellular loops, a linear pentapeptide sequence in the N-terminus (Fy6), and allelic N-terminal single nucleotide polymorphism (SNP) variants (FyA and FyB). Multiple ACKR1 phenotypes arise from SNPs in the upstream promoter and coding sequence of the *ACKR1* gene (28). The major isoform of ACKR1 is a 336 amino acid protein with two common alleles FyA (42Gly), FyB(42Asp), and the less common FyX, most associated with R89C (29).

**Figure 2:**
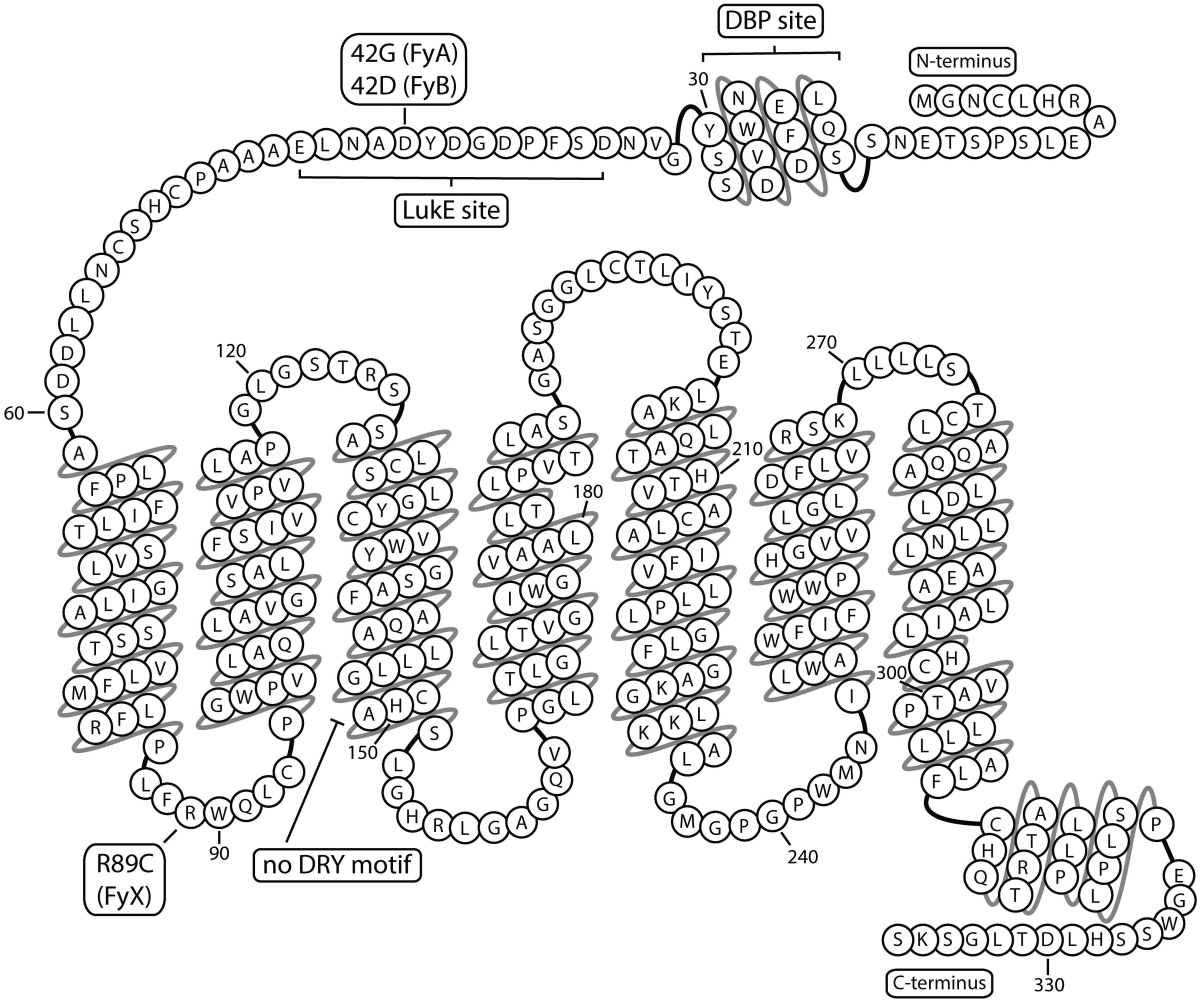
ACKR1 snake plot Atypical chemokine receptor 1 has seven transmembrane domains and multiple binding sites in the extracellular N-terminus. Residue 42 is depicted as aspartic acid corresponding to FyB variant. DBP, Duffy Binding Protein; LukE, Leukocidin E.

A unique selective pressure from malaria parasites contributes to distinct population-specific and geographic patterns of ACKR1 expression (30). The N-terminus of ACKR1 is a recognition site for *Plasmodium vivax* and *P. knowlesi*, which invade erythrocytes during blood infection (31). Malarial resistance is conferred by the “Duffy-negative” or “erythrocyte silent” (Fy^ES^) single nucleotide polymorphism (SNP), that alters the GATA1 transcription factor binding site in the *ACKR1* promoter, ceasing erythroid, but not endothelial, expression (32). The coevolutionary history of *Plasmodia* parasites and Fy^ES^ phenotype is complex, but the current evidence indicates that African *P. vivax* selected the “erythroid silent” polymorphism in the FyB allele in endemic regions. FyB^ES^ is now the prevalent phenotype of people in Africa, regions within the Arabian Peninsula, and with African ancestry (33, 34). The ancestral form of ACKR1 may have been FyB, which then adapted through the FyA variation (42G) conferring diminished susceptibility to *P. vivax* or the silencing polymorphism FyB^ES^ (rs2814778) (35, 36). The FyX variant is linked to both R89C and A100T mutations and decreases detection of ACKR1 expression (37). This effect may arise from a disruption in the first intracellular loop between the first and second transmembrane domains, and may interrupt trafficking to the membrane, impede protein folding, or cause formation of destabilizing inter/intra-molecular disulfide bonds (38, 39). The amino acid sequence of ACKR1 is depicted in **Figure 2**. Current understanding is that the primary drivers of differentiation of ACKR1 expression and the molecular basis of the Duffy blood group are the FyA/FyB alleles encoding Gly42 or Asp42 in the N-terminus and the Fy^ES^ SNP, which determines if ACKR1 is present on erythrocyte surfaces to display epitopes like Fy3 or Fy6. These genetic variations that alter ACKR1 expression and N-terminal sequence may have a significant impact on disease by changing the abundance and distribution of ACKR1 ligands (40, 41).

## ACKR1 structure and function

Chemokine receptors are activated after binding ligands in a multi-step interaction using the receptor N-terminus that extends from the first α-helical transmembrane domain. The chemokine binding pocket is formed within the transmembrane helices and the extracellular connecting loop regions. Engagement of a typical chemokine receptor triggers conserved microswitches and conformational changes in the transmembrane helices followed by activation of intracellular secondary messengers (42). G-protein coupling occurs at a conserved “DRYLAIV” sequence motif found at the intracellular end of transmembrane helix 3. However, atypical receptors have sequence modifications at this position that prevent G-protein mediated signaling. While ACKR1 has no homologous motifs at this position, ACKR2 has DKYLEIV, ACKR3 has DRYLSIT, and ACKR4 DRYVAVT. Another common feature of GPCRs is a feedback inhibition mechanism wherein sustained receptor activation leads to phosphorylation of the C-terminus by G-protein coupled receptor kinases (GRKs). GRK activity supports association with β-arrestins, causing receptor internalization and alternative signaling. Both CKRs and ACKRs have serine and threonine-rich sequences in the intracellular C-terminal domain that are substrates for GRK-mediated phosphorylation. β-arrestin recruitment has been described for ACKR2-4, but while ACKR1 has analogous sites encoded in the C-terminus, investigation of GRK interactions has yet to be thoroughly explored (43). Thus, ACKR1 with the lowest sequence similarity to the other chemokine receptors, seems to have a distinct activation mechanism and network of intracellular interactions that is distinct from other ACKRs (44–46).

Solved structures of chemokine receptors are limited in the resolution of receptor N-terminal interactions, but studies support the importance of this domain for atypical chemokine receptor function (47). The ACKR2 N-terminus is selective for CC-type chemokines, and a protein derived from the critical domains has been proposed as an anti-inflammatory chemokine sink (48). The N-terminus of ACKR1 is among the longest of any chemokine receptors and contains extended regions of amino acids modeled to form electrostatic interactions with the basic and positively charged surfaces characteristic of chemokines (49). A distinguishing feature of ACKR1 is the capacity to bind multiple CXC and CC class chemokines, and the flexibility of this mostly disordered region allows for variable configurations to dock many different ligands (50). The binding interactions at the N-termini of ACKRs are shown in **Table 1**. Discrete ACKR1 N-terminal residues determine ligand affinity and different segments have been successfully engaged by antibodies or antibody-derived fragments to prohibit ligand binding (51, 52). A chimeric construct with the N-terminus of ACKR1 and the transmembrane domains and extracellular loops of CXCR2 retained the binding profile of full-length ACKR1, with high affinity for non-CXCR2 ligands CCL5 and N-terminally modified CXCL1 (53). The independence of the N-terminus for certain ligands also suggests utility of a soluble platform with the binding affinity of ACKR1, for example as a decoy for pathogens targeting erythrocytes. Additional detailed structural data describing interactions between the ACKR1 N-terminus and different chemokine ligands will contribute to understanding conserved and chemokine-specific binding mechanisms.

**Table 1:** Ligands of atypical chemokine receptors 1-4.

	*CC*	*CXC*	*non-CK*
*ACKR1*	CCL2, CCL7, CCL11, CCL13, CCL14, CCL17 Weak*: CCL1, CCL8, CCL18	CXCL1, CXCL2, CXCL3, CXCL5, CXCL6, CXCL8, CXCL11, CXCL12, Weak*: CXCL9, CXCL10, CXCL13	LukE, HlgA, PvDBP, PkDBP
*ACKR2*	CCL2, CCL3, CCL3L1, CCL4, CCL4L1, CCL5, CCL7, CCL8, CCL11, CCL12, CCL13, CCL14, CCL17, CCL22	CXCL10	HIV gp120, Staphopain A
*ACKR3*	vCCL2	CXCL11, CXCL12	Adrenomedullin, Adrenorphin, BAM18/22, Dynorphin A/B, MIF, Nociceptin NH2, Peptide E
*ACKR4*	CCL19, CCL20, CCL21, CCL22, CCL25	–	–

Atypical chemokine receptors bind chemokines of CC and CXC classes and have non-chemokine ligands. ACKR1 is targeted by *Plasmodium vivax* and *Plasmodium knowlesi Duffy* Binding Proteins (PvDBP and PkDBP) and by *Staphylococcus aureus* toxin proteins Leukocidin E (LukE) and γ-hemolysin A (HlgA). *Chemokines demonstrated weak binding affinity to ACKR1 in competition assays and their physiological relevance is uncertain. ACKR2 has been reported to bind HIV envelope glycoprotein gp120 and is a substrate for *S. aureus* cysteine protease Staphopain A. ACKR3 binds numerous peptides, the peptide hormone adrenomedullin, endogenous opioid peptides in the dynorphin, enkephalin, and nociceptin families, and macrophage migration inhibition factor (MIF).-, none reported.

Initial surveys of ACKR1 functions suggested a binding preference for chemokines containing the sequence motif “ELR” in the N-terminus, a subgroup of CXC chemokines distinguished for its capacity for angiogenesis and inflammatory signaling through neutrophil receptors CXCR1 and CXCR2 (54, 55). One of the first reported angiogenic chemokines was CXCL8, and a model of neovascularization emerged with ELR^+^ CXCR2 ligands stimulating endothelial migration and tube formation countered by ELR^-^ CXCR3 ligands. Angiogenic effects have since been ascribed to non ELR^+^ CXCL12 and other CC chemokines, particularly CCL2, suggesting a multifactorial system of CXC and CC chemokine receptors on endothelial cells and other immune cell types (56, 57). Evidence for the anti-angiogenic properties of ACKR1 was initially shown in a mouse by overexpressing ACKR1, decreasing CXCR2-mediated corneal angiogenesis in response to CXCL2 stimulation (58). Further investigation using radioligand displacement supported strong binding of ACKR1 to ELR^+^ chemokines like CXCL5 and CXCL8 that signal through CXCR2, but highest binding affinities were calculated for CCL5, CCL7, and non-ELR^+^ CXCL11 (59). The next functional categorization was regulation of “inflammatory” chemokines over “homeostatic” chemokines since chemokines CXCL12 and CCL21 showed weak ability to displace CXCL8 bound to ACKR1 (59). However, studies have since described many roles for both chemokines in inflammation and binding interactions have been reported between ACKR1 and CXCL12 (60, 61). ACKR1 binds most chemokines including the ELR^+^ CXC subfamily, and chemokines CXCL10, CXCL13, and CCL1 that were reported as non-binders were found to have weak but sub-micromolar affinities for ACKR1 on human erythrocytes (59). ACKR1 does not bind every chemokine, for example CXCL4 and several lymphoid CC chemokines have been shown not to bind ACKR1-expressing cells (59, 62).

The binding profile of ACKR1 has been primarily surveyed using radioligand displacement assays with pre-bound, high-affinity ligands and erythrocyte ACKR1 that may underrepresent lower-affinity interactions with chemokines or the influence of other mediators on endothelial surfaces like glycosaminoglycans. This selectivity was reported to play a role in filtering chemokines at high endothelial venules (HEVs), where ACKR1 may restrict inflammatory chemokines from entering secondary lymphoid organs and interfering with chemokine sensitivity (62).

While ACKR1 is most readily detected on mature erythrocytes, ACKR1 expression is highest in the bone marrow on progenitor nucleated erythroid cells (NECs), where key cell contacts are made with hematopoietic stem cells (HSCs) (63). The erythroid silent variant (FyES), though providing malarial protection, loses this developmental cue, resulting in a neutrophil phenotype with altered surface markers and increased propensity to leave circulation (64, 65). The observed neutropenia, historically called “benign ethnic neutropenia” and now more accurately “Duffy-associated neutrophil count” (DANC), does not eliminate effective inflammatory immune responses and is hypothesized to be asymptomatic in otherwise-healthy patients (66–68).

Outside of the erythroid lineage, ACKR1 is expressed on endothelial cells of post-capillary venules, where affinity for certain chemokines results in immobilized gradients that direct cell migration (69–71). A hallmark of tissue inflammation is increased chemokine production, but chemokines must be concentrated and displayed in the vascular compartment with a coordinated gradient to effectively direct immune responses. Endothelial ACKR1 function involves a combination of chemokine retention, presentation to circulating leukocytes, and trafficking from tissues to the luminal surface (72). ACKR1 is distinguished from the other ACKRs by ligand-triggered chemokine transcytosis through venular endothelial cells. ACKR1 has been shown to transport chemokines from basolateral to luminal sides of endothelial cells and retain chemokines on the apical surface promoting signaling through GPCRs (73–76). One demonstration of this function is neutrophil diapedesis, where ACKR1 concentrated at endothelial junctions binds and exchanges CXCL1 and CXCL2 chemokines to direct neutrophils and prevent reverse migration (77). These functions at the endothelium have been shown to modulate neuroinflammation as well, by trafficking chemokines and immune cells across the blood-brain barrier (78, 79). ACKR expression is detected in the brain on cerebellar Purkinje cells, where it may regulate cellular excitation for smooth motor control (53, 80). Further studies of ACKR1 in different tissues, including neurons, and with non-chemokine ligands may reveal additional complexity and specialized functions.

## ACKR1 and infectious disease

The extracellular domain of ACKR1 is a potential target to inhibit pathogenicity mechanisms of atypical malaria, *S. aureus*, and HIV. *Plasmodia* malarial parasites replicate and mature inside human reticulocytes and erythrocytes, and the “atypical” *P. vivax* and *P. knowlesi* parasites identify these targets by secreting Duffy Binding Protein (DBP), which binds to and oligomerizes around the N-terminal domain of ACKR1 (81). While *P. falciparum* secretes multiple soluble factors, atypical malaria invasion can be avoided with the erythroid silent polymorphism or by blocking the DBP-ACKR1 binding interface with inhibitory chemokines or antibodies (51, 82, 83). Crystal structures have been solved showing a dimer of PvDBP dimers binding a peptide corresponding to ACKR1 residues 14-43. The receptor peptide could be resolved between residues 19-30 as an amphipathic α-helix structure with Y30 oriented towards a positively charged pocket (84). An ACKR1 mimetic was designed from this N-terminal helix, with the DBP-binding residues grafted onto a stable scaffold (85). The engineered protein could successfully inhibit DBP dimerization and binding to erythrocytes. Non-*falciparum* malaria, particularly from *P. vivax*, is an increasingly widespread disease that can cause severe or fatal illness, and the dependence on ACKR1-mediated invasion provides a prime therapeutic target (86).

A role for ACKR1 has been proposed in HIV pathogenesis, however the potential mechanisms of interaction are unclear. HIV uses chemokine receptors CXCR4 or CCR5 as co-receptors for targeting leukocytes, and the CCR5 inhibitor Maraviroc can successfully prevent binding by viral glycoproteins (87). Some studies have proposed ACKR1 is involved in HIV interactions with erythrocytes that promote infection of other blood cells or maintain a viral reservoir (88–90). However, the FyES phenotype was not confirmed to alter HIV susceptibility or disease progression (91, 92).

ACKR1 is also a target for *Staphylococcus aureus* toxins LukED and HlgAB (93). *S. aureus* bacteremia is particularly dangerous because these pore-forming, bicomponent toxin systems cause hemolysis and vascular leakage when they engage ACKR1 on red blood cells and endothelial junctions (94, 95). A crystal structure of the LukE toxin protein and the ACKR1 N-terminus resolved residues 34-46 of the receptor with Y41 stabilized in a lysine and arginine-enriched viral pocket, similar to the mechanism of interaction observed in the crystal structure of PvDBP and ACKR1 (96). Further analysis using time-resolved mass spectrometry and resonance energy transfer from a C-terminal bioluminescent tag suggests toxin binding may modulate receptor conformation to form ACKR1 homodimers and even alter interactions with intracellular G_αi_1 subunits (97). Structure-guided strategies targeting ACKR1 could be useful to address pathogenicity mechanisms of significant infectious agents.

## ACKR1 and pathoinflammation

Immune dysregulation involves an excess of chemokines and other soluble inflammatory mediators and can incur tissue damage from resultant immune cell infiltrates. Modulation of the chemokine network to treat autoimmune disease has yielded promising leads, but few have shown clinical effectiveness and safety (98, 99). Currently trials are ongoing for a CCR9 antagonist for Crohn’s disease and a CCR1 antagonist for rheumatoid arthritis (100, 101). Reparixin, an allosteric CXCR1 and CXCR2 blocker, did not progress past a phase 3 trial as a drug adjuvant for pancreatic islet allotransplantation to treat type 1 diabetes, but it is still a candidate for ongoing trials for metastatic breast cancer and COVID-19 related acute lung injury (102–104). Alternatively, blocking chemokines may decrease autoinflammation, and an antibody drug bertilimumab targeting CCL11 was designed to prevent eosinophil-mediated autoimmune damage in bullous pemphigoid skin disorder and inflammatory bowel disease (105, 106). Administration of anti-CXCL10 antibody was a promising strategy to limit cytotoxic T-cell liver damage, but clinical utility was hindered by continuous CXCL10 secretion and retention on endothelial cells (107, 108).

Controlling chemokine concentrations *via* ACKR1 could contribute to the success of these drug strategies or offer new avenues for regulating immune responses. ACKR1 regulation may contribute to resolution of chemokine-driven inflammation. ACKR1 binds chemokines at the inflamed synovial endothelium, and diminished expression of ACKR1 may be associated with rheumatoid arthritis (109). People with the FyES phenotype that decreases erythrocyte ACKR1 were observed to have increased IgE in serum samples and higher susceptibility for asthma (110). Knocking out all ACKR1 expression in an endotoxin-induced mouse model of inflammation was shown to increase lung and liver damage from granulocytic infiltrates (111). These studies support a protective role for ACKR1 by decreasing circulating chemokine levels, particularly through expression on erythrocytes.

However, ACKR1 on the endothelial surface may have separate functions in chemokine retention and has been observed to increase leukocyte recruitment and activity (112). Endothelial ACKR1 expression may potentiate respiratory distress, as seen in patients with suppurative pneumonia, and require balance from erythrocyte ACKR1 to avoid acute lung injury (113, 114). This finding has been reinforced in mouse models of lung inflammation, where studies show that ACKR1 knockout mice are protected from neutrophil-mediated tissue damage (115, 116). ACKR1 receptors supporting chemokine-mediated leukocyte infiltration have also been reported to contribute to patient lesions of giant cell/temporal arteritis and nephrotoxicity in a mouse model of renal failure (117, 118).

ACKR1 can also facilitate neutrophil reverse transendothelial migration and indirectly cause systemic inflammation (119). Using aged mice subjected to IL-1 stimulation, ACKR1 was shown to concentrate mast cell derived CXCL1 at endothelial junctions, causing desensitization of CXCR2 on circulating neutrophils and dysregulated chemotaxis. Without tight regulation of chemokine patterns, the activated neutrophils migrated to the lung leading to vascular leakage, which could be a targetable mechanism for aging-related inflammation or acute lung injury such as COVID-19 pneumonia (120, 121). An increase in ACKR1 expression was also detected in humoral and cellular rejection of renal allografts, but it remains unclear if upregulation is induced by an inflammatory program, or which component of graft rejection would be influenced (122, 123).

Chemokines are also important mediators of chronic inflammatory damage in cardiovascular disease, including atherosclerosis, where chemokine concentrations, combinations, and oligomerization all contribute to initiation and progression of vascular lesions (124). ACKR1 involvement and targeting to treat atherosclerosis was initially proposed because endothelial dysfunction and chemokines like CXCL8 immobilized on erythrocyte membranes contribute to plaque formation and coronary artery disease (125, 126). In an atherosclerosis mouse model, knocking out ACKR1 led to diminished plaque formation, cellular infiltrate in the vessel walls, and activation of macrophages (127). As the chemokine network is further studied in the context of cardiovascular diseases, ACKR1 binding inflammatory chemokines may become a relevant drug target. More detailed investigation is required to discern the role of ACKR1 in acute and chronic phases of inflammation and what changes in cellular immune responses may be feasible by targeting ACKR1.

## Cancer angiogenesis, metastasis, prognostics

Therapeutic cancer interventions include drugs to attack primary tumors or alter pro-metastatic signals and biomarkers for prognostic screening. Chemokine patterning and chemokine receptor signaling are integral to the proliferation and spread of tumor cells (128). A challenge to targeting CKRs in cancer is that the same chemokines that stimulate tumor growth and neovascularization can also activate and direct tumor-killing immune cells. For example, CCL5 signaling through CCR5 supports recruitment of anti-tumor natural killer cells and cytotoxic T cells, but also stimulates pro-tumor, tissue-resident myeloid cells and lymphocytes (129). Nevertheless, the chemokine receptor drugs that have demonstrated promising anti-cancer activity in clinical trials, particularly antagonizing CCR2, CCR4, CXCR2, and CXCR4, emphasizes the importance of studying chemokine regulation and receptor mechanisms (130).

Neovascularization of an emerging tumor is an essential process to tumor growth and vascular access that involves distorting the balance of pro and anti-angiogenic chemokines (131). Angiogenesis is difficult to target because it can be triggered by tumor cells through an increase in CXCR2 agonism, or by a change in the cellular tumor infiltrate that favor tumor-associated macrophages (132). The mechanism of ACKR1 regulating pro-cancer chemokine signaling involves interplay between endothelial cells and erythrocytes that influences the activation of GPCRs CXCR2 and CXCR3. ACKR1 and the ACKR subfamily may balance chemokine abundance and patterning to benefit host immune cell recruitment that is lost in unregulated, aggressive cancer types (133, 134).

**Figure 3:**
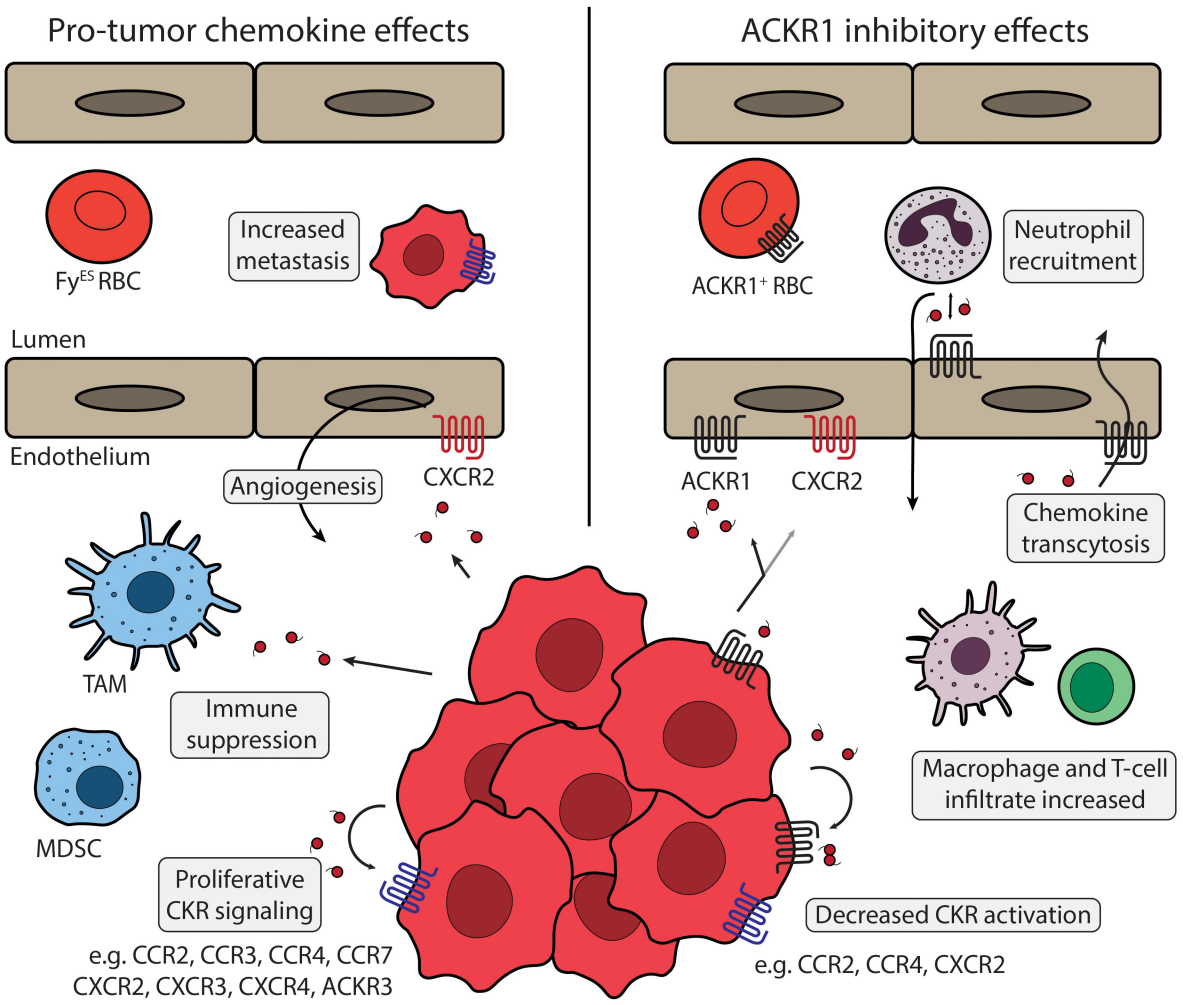
ACKR1 and tumor microenvironment Chemokine signaling in the tumor microenvironment is regulated by ACKR1 expression. Left panel describes chemokine effects that promote tumor phenotypes. ACKR1 (black) expression can be diminished on tumor cells or by the Fy^ES^ polymorphism. Angiogenesis can be triggered by chemokines secreted from TAMs, stromal cells, or by cancer cells themselves *via* activation of endothelial CXCR2 (red). Cancer cells release numerous chemokines, including CCL2, CCL5, CXCL8, and others that can act to suppress anti-tumor immunity. Various cancer types express a panel of CKRs (blue) including CCR1, CCR2, CXCR2, CXCR4, and others that support tumor proliferation and metastasis. Primary tumors can silence expression of chemokines like CXCL12 and increase expression of CKRs like CXCR4 to promote metastasis. Right panel shows proposed mechanisms of ACKR1 regulation. ACKR1 receptors on erythrocytes can act as a sink to buffer chemokine levels and may have interactions with ACKR1 expressed on endothelial cells. ACKR1 enrichment at endothelial junctions promotes neutrophil diapedesis *via* CXCL1 and CXCL2 exchange, and increased endothelial ACKR1 improves recruitment of macrophages, CD4+ and CD8+ T-cells. Expression of ACKR1 in cancer models or patient tumor samples has been shown to modulate CCL2 and CXCL8, ligands of CCR2, CCR4, and CXCR2. ACKR1 modulates many chemokines and regulation of multiple CKRs may contribute to the improved clinical outcomes observed. TAM, Tumor associated macrophage; MDSC, Myeloid-derived suppressor cell; Fy^ES^ RBC, “Erythroid-silent” erythrocyte; CKR, chemokine receptor.

Studies show that when ACKR1 is expressed on malignant cells it is protective against tumor angiogenesis and subsequent metastasis. Proposed contributions of ACKR1 are shown in **Figure 3**. When transgenic ACKR1^+^ non-small cell lung cancer cells were implanted in SCID mice, the resulting tumors had decreased vascularization, and metastatic potential (135). Immunoassay for chemokines secreted by ACKR1^+^ tumor cells showed a decrease in CXCL5 and CXCL8, and chemokine detection suggested the chemokines were bound by ACKR1 and internalized or immobilized on the cell surface rather than removed from the tumor microenvironment. Another study injected mice with different cancer cell lines that expressed high or low levels of ACKR1 levels to show that cancer invasiveness was inversely related to ACKR1 activity (136). MDA-MB-231 breast adenocarcinoma cells were used to represent aggressive breast cancer with low endogenous ACKR1 expression, and MDA-MB-435 melanoma cells were used to model an ACKR1-expressing tumor (137, 138). Testing in either cell culture or the tumor xenografts showed that ACKR1 expression could prevent the spike of CCL2 and CXCL8 released into the growth media or tumor microenvironment. These findings were correlated with a breast cancer clinical cohort, where patients with higher levels of detectable ACKR1 had less invasive cancers and lower mortality rates. Altering the global ACKR1 expression also changes the tumor microenvironment. ACKR1 global knockout in a spontaneous murine prostate cancer model resulted in less dense, more necrotic tumors with increased intratumor concentrations of CXCL1 and CXCL2 (139). Overexpression of the endothelial ACKR1 in mice implanted with melanoma tumors demonstrated inhibition of tumor growth and vascularity and showed an increase in CD4^+^ and CD8^+^ T-cell and macrophage infiltration (140).

Angiogenesis is a continual process in healthy tissue that involves migration, proliferation, and differentiation and ACKR1 could influence feedback mechanisms triggered by CXCR2 signaling pathways. A study investigated how ACKR1 expression on non-malignant endothelial cells could decrease capillary formation and detected an upregulation of senescence biomarkers (141). In pancreatic cancer cells lines, co-expression of ACKR1 in CXCR2+ tumors was sufficient to inhibit CXCL8-triggered activation of STAT3 and mediators of epithelial-mesenchymal transition (142, 143). Blocking these oncogenic pathways is an important strategy to induce cellular senescence and restore the anti-tumor effects of immune defenses (144, 145). CXCR2 has a complex role in tumor progression, as receptor overstimulation and autocrine activation may also trigger and sustain a p53-mediated cellular senescence (146). Furthermore, it is possible ACKR1 could contribute cell cycle regulation through other interactions including the tumor suppressor CD82/KAI1, a multifunctional surface tetraspanin. A study found that CD82^+^ cancer cells have increased adherence to ACKR1^+^ vascular endothelial cells and suggested that a direct interaction leads to p21 cyclin-dependent kinase inhibition and prevention of metastatic escape (147). A follow-up study also detected p21 upregulation connected to CD82 and potentially ACKR1, and implied that CD82 opposes CXCL8 effects by downregulating secretion from tumors and displacing CXCL8 from endothelial ACKR1 (148). The data interpretation from these reports is limited without testing CXCR2 signaling or reliable antibody detection of ACKR1.

Another important target of anti-cancer therapeutics is metastasis, the major cause of cancer mortality (149). Blocking chemokine signaling is an appealing strategy because metastatic invasion of susceptible cellular niches is inefficient without chemokine-directed migration and often characterized by chemotactic GPCR overexpression (150). ACKR1 may play a role in fine-tuning the complex chemokine patterns that are hijacked by migrating cancer cells. Many of the studies that observed an inverse correlation between the proliferative potential of primary tumors and ACKR1 expression also reported a decrease in metastatic phenotype. Another possible mechanism is alteration of the chemokine oligomeric equilibrium. Chemokine dimers elicit distinct signaling from monomeric chemokines, potentially representing feedback inhibition that could be used as an antimetastatic cue (5, 151, 152). Multiple factors increase the propensity of chemokine dimerization, including GAGs and interactions with the N-termini of GPCRs (153, 154). ACKR1 also shows similar activity by binding preferentially to the dimeric form of CXCL12 (155). Improved quantitation of chemokine concentrations in different cellular compartments and the relation between dimerization and chemotaxis are needed to predict the effects of ACKR1 preferentially binding certain chemokines as dimers.

Testing ACKR1 genotype and expression in tumor biopsies may be a clinically useful cancer biomarker. Multiple studies have indicated that higher ACKR1 expression levels in breast cancer tumors improve relapse-free patient survival, while loss of ACKR1 expression, frequently in patients with African ancestry, is an indicator of increased tumor aggressiveness, metastatic propensity, and mortality (156–162). Detailed analysis is warranted for different cancer types, since comparing prostate cancer incidence within patient groups did not detect a strong correlation between the FyES polymorphism and increased cancer risk (163, 164). Additionally, blood typing to discern ACKR1 phenotype could be an effective, low-cost way to inform cancer treatment. ACKR1-mediated DANC neutropenia affects patient care by impeding administration of drugs like clozapine or azathioprine and leading to potentially unwarranted bone marrow biopsies (165–167). Patients with FyES phenotype are at increased risk of side effects from chemotherapy but using the same neutropenic cutoff values may unnecessarily delay initiation and prolong duration of cancer treatment (168–172). Adapting standard of care for patients with DANC could provide an opportunity to address disparate treatment outcomes with a precision medicine approach. Overall, a cancer-protective role for ACKR1 is supported by cell culture, mouse models, and genetic associations, and independent anti-angiogenic properties for endothelial, erythroid, and tumor ACKR1 expression can contribute to improved patient outcomes.

## Discussion

ACKR1 exhibits favorable structural features, expression profile, and biological activity for development of therapeutic interventions. More investigation is needed to determine the extent of control over chemokine scaffolding by ACKR1 that can be attained by different classes of molecules. Antibodies binding to different ACKR1 epitopes do not uniformly inhibit chemokine binding, suggesting some capacity to alter ACKR1 specificity. Development of screening readouts for binding that can supplement competition assays will facilitate identification of small molecules. The independence of chemokine-binding and DBP recognition sites located in the extended N-terminus indicates that this domain could be isolated to provide an effective ACKR1 decoy, similar to a strategy proposed for the ACKR2 N-terminus. The positioning and functions of ACKR1 receptors in the hematopoietic compartment, on the surface of erythrocytes, and at the junctions of endothelial regions specialized for cell trafficking provide an opportunity to control immune cell migration into tissues. Additionally, further exploration of the impact of ACKR1 expressed at the blood-brain barrier and on different neuronal cell types may reveal a targetable role in regulating neuroinflammation. Still, the mechanisms of ACKR1 retaining or sequestering different chemokines have yet to be elucidated in detail, particularly in the context of the tumor microenvironment. Assigning ACKR1 expression to specific cell types within and around tumors of different origins will be needed to understand the correlation observed in experimental models between ACKR1 expression and decreased malignant phenotypes.

Targeting ACKR1 is an appealing approach for new compounds that modulate chemokine biology without interfering with the chemokine sensitivity and signaling functions of immune cell CKRs. ACKR1 in circulation is only reliably found in post-capillary venules and erythrocytes rather than myeloid or lymphoid cells, suggesting targeting ACKR1 would not directly impact immune effector function. While some studies report ACKR1 detection on other cells like bone marrow macrophages, these reports use a polyclonal antibody which has been shown to recognize non-ACKR1 surface markers (173, 174). Furthermore, unlike the other ACKRs, ACKR1 functions seem independent of G-protein or β-arrestin signaling pathways (175). The restricted tissue and signaling capabilities suggest side effects of ACKR1 inhibition may be modest compared to the signaling GPCRs or other ACKRs. As ACKR1 biology and molecular pharmacology are examined in greater detail, development of new ligands to alter its function will be useful as research tools and may enable amelioration of specific disease pathologies.

Current opportunities for intervention should include shielding extracellular ACKR1 residues from virulence factors of important human pathogens. This approach may have multiple benefits, including preventing erythrocytic replication of *Plasmodia* and maintaining the integrity of endothelial junctions during *S. aureus* infections. Additionally, animal models, cancer cell experiments, ACKR1 biochemistry, and meta-analysis of clinical cohorts all indicate ACKR1 activity impedes cancer progression. This underscores the importance of elucidating ACKR1 chemokine-binding mechanisms and the impact on immune cell responses to tumors to take steps towards enhancement or reconstitution of ACKR1-mediated protection in cancer therapy. Until then, ACKR1 may be used as a prognostic indicator for the aggressiveness of different cancer types and may be inform treatment regimens for patients with different patterns of ACKR1 expression. The next steps include detailing the binding interactions of different chemokines to ACKR1 and the mechanisms that alter receptor expression and enable chemokine trafficking through cells. Future development and implementation of therapeutics that target the chemokine network should consider the role of ACKR1 in patient physiology and the possibility of targeting ACKR1 itself.

## Author contributions

Manuscript written and edited by KC and BV. All authors contributed to the article and approved the submitted version.
